# Impact of polystyrene microplastic exposure at low doses on male fertility: an experimental study in rats

**DOI:** 10.1038/s41598-026-38385-y

**Published:** 2026-02-23

**Authors:** Aisha H. A. Alsenousy, Asmaa Hassan Younis Khalaf, Hesham Zaki Ibrahim, Maher A. Kamel, Mokhtar Ibrahim Yousef

**Affiliations:** 1 Department of Biochemistry, Medical Research Institute, University of Alexandria, 165 El-Horeya Rd, Al Ibrahimeyah Qebli WA Al Hadrah Bahri, Qesm Bab Sharqi, Alexandria, 21561 Egypt; 2 Department of Environmental Studies, Institute of Graduate Studies and Research, Alexandria University, Alexandria, Egypt

**Keywords:** Polystyrene microplastics, Reproductive toxicity, Reproductive hormones, Molecular parameters, Semen characteristics, Oxidative stress, Histological changes, Biochemistry, Environmental sciences, Physiology

## Abstract

Polystyrene microplastics (PS-MPs), widely used in commercial and pharmaceutical products, are emerging endocrine-disrupting pollutants with potential reproductive toxicity. This study evaluated the dose-dependent effects of PS-MPs on adult male rats by assessing semen quality, reproductive hormones, oxidative stress, mitochondrial and inflammatory markers, and testicular histology. Rats were assigned to six groups: a control group and five groups receiving PS-MPs orally (0.1, 1, 10, 20, or 40 µg/kg BW) for 45 days. PS-MP exposure reduced sperm count and motility, increased abnormal sperm, decreased testosterone, and elevated FSH and LH. Mitochondrial biogenesis/function markers (PGC-1α, UCP1, TFAM) were downregulated, while NF-κB, caspase-3, and TBARS were increased, accompanied by significant depletion of antioxidant defenses (GSH, GR, GPx, SOD, GST, CAT, TAC) and pronounced testicular histopathology. These effects were dose-dependent, and PS-MPs were detected in testicular tissue by pyrolysis-GC/MS at the doses of 10 µg/kg and higher. Collectively, the data identify mitochondrial dysfunction–driven oxidative stress and associated inflammation as a key mechanism by which PS-MPs induce spermatogenic failure, hormonal disruption, and testicular damage, highlighting their potential as potent male reproductive toxicants.

## Introduction

Microplastics (MPs) are defined as plastic particles smaller than 5 mm. They result from the physical, chemical, and biological degradation of larger plastic items. Given their ubiquity and potential toxicity, MPs have garnered significant attention as a major environmental contaminant. Of particular concern is their impact on male fertility; MPs have been observed to accumulate in mammalian reproductive organs, leading to documented negative effects on sperm quality. Therefore, understanding the precise effects of MP exposure on the male reproductive system and sperm remains a crucial research priority^1,2^. The majority of MPs have been found in rivers, sewage, sediments, urban beaches, and oceans^3^. Microplastics, when ingested, inhaled, or absorbed through the skin, are capable of causing oxidative stress, inflammation, endocrine disruption, and cellular damage. These effects collectively represent a significant hazard to the male reproductive system’s health and function^4^.

Microplastics have been discovered to have negative impacts on aquatic and mammalian species, lowered body condition and feeding activity, neurotoxicity, inflammation, oxidative damage, intestinal barrier malfunction, and disruption of energy^5^. According to earlier research, MPs’ toxicity in many tissues is directly correlated with their particle size. An organism’s reproductive system is vital to its existence. MPs are reproductively harmful to aquatic species, including Daphnia and Hydra attenuata^6^, medaka fish^7^, and oysters^8^. Researchers have shown that after acute and short exposure to MPs, testes exhibited oxidative damage, abnormal levels of hormones, and reduced sperm quality^9^.

A variety of materials, including polyvinyl chloride (PVC), polyethylene (PE), and polystyrene (PS), are used to create microplastics. Polystyrene (PS) is one of the most commonly utilized aromatic monomers^10^. MPs made from polystyrene pose a major environmental risk and are referred to as “white pollutants.”^11^. Three main routes exist for polystyrene microplastics to enter the human body: oral intake, which includes drinking water^12^, marine-based products^13^, and other food products^14^. The other two are breathing^15^ and skin contact^16^.

Microplastic particles found in toothpaste, synthetic clothes, and facial cleansers enter the aquatic environment through home and commercial drainage systems and wastewater treatment plants^17^. Recently, MPs have been identified in food, air, and water used for drinking^18^. Previous studies suggested that the size and form of the MPs may influence their elimination efficiency^19^. The risk increases with decreasing microplastic particle size^6^. At the biological and molecular level, polystyrene exposure leads to the development of inflammatory responses and oxidative damage in the crab liver^20^.

The total number and the diameter of oocytes, as well as the quantity of sperm and offspring oysters, can all decrease after being exposed to PS-MPs^8^. Additionally, it may disrupt the medaka’s hypothalamic-pituitary-testicular axis, which could lead to aberrant blood levels of sex hormones and reproductive endocrine abnormalities^21^. Furthermore, Sussarellu et al. revealed that exposure to PS-MPs disrupted oyster reproduction, particularly affecting the larval stages. PS-MPs can elevate inflammatory markers such as TNF-α and IL-6 in the embryos of zebrafish^22,23^. Exposure to MPs has been shown in other research to disrupt spermatogenesis and gonadotropin-releasing hormone (GnRH) levels in male rats, and to induce both metabolic abnormalities and generational abnormalities in mice^24^. Exposure to PS-MPs in male mice resulted in an increased rate of sperm malformation and a subsequent reduction in the amount of viable epididymal sperm, ultimately leading to testicular irritation^25^. Furthermore, Hou et al. (2021) discovered that PS-MPs may induce apoptosis and pyroptosis in ovarian granulosa cells. These findings suggested that therapy with PS-MPs might cause reproductive system malfunction. Mice exposed to PS-MPs via drinking water or gavage exhibited impairments in the reproductive system, including decreased spermatogenesis and disruption of the blood-testis barrier^26^.

According to recent research, MPs can decrease both the quality and amount of sperm in mice. Meanwhile, a different study has demonstrated that PS-MPs can penetrate mice’s testes, rupturing the Blood-Testis Barrier (BTB)^26^. Male infertility is a major issue in human reproduction, with male factors accounting for around half of cases. Over the past 80 years, male semen quality and quantity have drastically decreased for unclear causes, with pollution being one of the primary hypotheses^27^. The reproductive damage caused by PS-MPs, size 5 μm, at various doses is not well documented in prior investigations. Thus, this research aims to investigate the impact of multiple dosages (from 0.1 to 40 µg/kg B.W.) of PS-MPs on the reproductive system of adult male rats via semen characteristics, reproductive hormones, molecular parameters, oxidative stress, and testes histological changes. Also, PS-MPs were present in the testes using pyrolysis-gas chromatography/mass spectrometry.

## Materials and methods

### Tested compounds and doses

Monodisperse polystyrene microplastics (PS‑MPs) were purchased from Tianjin Baseline ChromTech Research Centre (Tianjin, China; Product No. 6-1-0 50 0; 10 ml, 5% w/v). According to the manufacturer’s datasheet, the particles are monodispersed spherical (microspheres) polystyrene microspheres with a narrow size distribution centered around 5 μm. The hydrodynamic size distribution and zeta potential of PS‑MPs were determined by dynamic light scattering (DLS) using Malvern Zetasizer Ultra (Malvern Instruments, UK) at 25 °C. Suspensions were prepared in distilled water and sonicated for 30 min before measurement. The mean hydrodynamic diameter, as well as the zeta potential, was recorded from three independent measurements.

The doses of polystyrene microplastics (PS-MPs) were 0.1, 1, 10, 20, and 40 µg/kg B.W. The doses were chosen according to^22,28,29^. PS‑MPs were dispersed in distilled water to form a homogeneous suspension. The required mass of PS‑MPs was weighed and suspended in distilled water by vigorous vortexing and brief sonication to minimize aggregation before oral gavage. All reagents and chemicals were of analytical grade.

### Chemicals

All chemicals were of the highest quality. Reduced glutathione (GSH), nicotinamide adenine dinucleotide phosphate (NADPH), thiobarbituric acid RNA binding columns, homogenization columns, buffer TR, inhibitor removal buffer, wash buffer, DNase I, digestion buffer, digestion enhancer, RNase-free water, proteinase K, 80% ethanol, absolute ethanol, mercaptoethanol, glacial acetic acid, EDTA, loading dye solution, bromophenol blue, glycerol, ethidium bromide, and almost all other chemicals were purchased from Sigma Chemical Company (Saint Louis, USA).

### Polystyrene microplastics detection by pyrolysis-gas chromatography/mass spectrometry

Pyrolysis–GC/MS (Agilent MassHunter B.07.04) was used to semi-quantitatively confirm the presence or absence of PS‑MP–derived pyrolysis products in testicular tissue. The gas chromatograph is equipped with a flame ionization detector (FID) and has a sensitivity of 0.01 µg/kg. The gas chromatograph must be capable of linear temperature control from 50 °C to 320 °C for the capillary column oven. The gas chromatograph must be capable of controlling multiple valve events. Carrier gas flow controllers and or electronic pressure control modules shall be capable of precise control where the required flow rates are low. Pressure control devices and gauges shall be capable of precise control for the typical pressures required. The temperature program rate must repeat to within 0.1 °C and provide retention time repeatability of 0.05 min throughout the temperature program. Pre-Column Column-WCOT Column, 25 m long by 0.53 mm inside diameter fused silica WCOT column with a 1.0 micron film thickness of polydimethylsiloxane or any column with suitable chromatographic resolution. Analytical Column-WCOT Column, 100 m by 0.25 mm inside diameter fused silica WCOT column with a 0.5 micron film thickness of polydimethylsiloxane or any column with suitable chromatographic resolution as presented in Table 1.

**Table 1:** Gas chromatograph-mass spectrometer protocols for the detection of polystyrene microplastics.

Gas chromatograph	
Inlet Type:	Programmable split/splitless
Inlet Temperature:	200 °C for 14 min, then 200 °C/Amin to 400 *C, hold until end of run
Oven temperature program:	50 °C for 2 min, then 7 °C min to 200 °C for 0 min
Run Time:	24 min
Columns:	30 m, 0.25 mm ID, 0.25 μm film polydimethyl siloxane (pre-column); 60 m, 0.32 mm ID, 0.5 μm film 5% phenyl polydimethyl/siloxane (analytical column)
Bypass restrictor:	2 m × 0.150 pm deactivated fused silica tubing
Carrier gas:	Helium
Carrier gas:	PSS Inlet Pre-column: 38 psig (261 kPa) for 14 min then 2 psig (13.78 kPa) for the remainder of the run
Carrier gas:	Split flow rate 25 mL/min for duration of run
Carrier gas:	Analytical Column - Auxiliary Pressure Module set at 15 psig (103.4 kPa) for the entire run
Mid-point monitor detector:	Flame ionization
Mass spectrometry	
GC-MS Interface:	Direct
GC-MS Interface	250 °C
Temperature:	Quadrupole mass spectrometer
Detector:	Full scan with extracted ion quantification
MS Data Acquisition Mode:	(eV) 70 fixed operating condition
Ionization Voltage: Mass Scan Range: MS source Temperature:	m/2 35–200 230 °C

Mass spectrometry is capable of producing electron ionization spectra at 70 electron volts or higher and is capable of scanning the range of the specified quantitation masses or (m/e). The mass scan range shall cover the masses of interest for quantitation and should yield at least 5 scans across the peak, as presented in Table 1. The method was optimized to detect styrene monomer and trimer peaks characteristic of polystyrene, but was not calibrated to provide absolute PS‑MP concentrations in this study.

### In vivo study

The in vivo experiments were conducted on thirty male Wistar Dawley rats (adults) of 9–12 weeks of age and weighing 180 ± 5 g obtained from the Faculty of Medicine, Alexandria University, Egypt. The research was conducted in accordance with the Guide for the Care and Use of Laboratory Animals (International Council for Laboratory Animal Science, ICLAS). It was approved by the local ethical guidelines of the Institutional Animal Care & Use Committee (IACUC) at Alexandria University, Egypt (Approval No. AU14-248-18-2-11). All methods were performed in accordance with the guidelines and regulations of the same committee. All rats were provided *ad libitum* with a standard commercial rodent chow diet, containing approximately 9% fat, 20% protein, 53% starch, 5% fiber throughout the experimental period. Animals were kept in normal atmospheric conditions at a temperature of 25 ± 5 °C, and 50–60% humidity was maintained throughout the experiments with a 12 h light/day cycle.

### Experimental design

Animals were divided into 6 equal groups of five rats each as follows: group 1 served as the vehicle control and received distilled water by oral gavage (same volume as treated groups) once daily for 45 days, and groups from 2 to 6 were administered polystyrene microplastics at doses of 0.1, 1, 10, 20, and 40 µg/kg B.W. Animals were orally gavage-administered with respective doses every day for 45 days. At the end of the 45‑day exposure, all rats were deeply anesthetized with isoflurane, euthanized by cervical dislocation, and subjected to necropsy for the collection of blood and reproductive organs. No animals were kept alive after completion of the experiment. Carcasses and remaining tissues were disposed of in accordance with the biosafety and animal facility regulations of Alexandria University.

### Samples Preparation

Blood samples were collected into tubes containing heparin as an anticoagulant and then centrifuged at 860 × g for 20 min to separate the plasma, which was stored at -80 °C until analysis of the testosterone, FSH, and LH. Testes divided into two aliquots: (i) for the extraction of total RNA for quantitative real-time polymerase chain reaction (qRT-PCR) analysis, to assess the gene expression of PGC-1α, mt-TFAM, and UCP-1, (ii) for protein assays as the testes were minced and homogenized (10%, w/v), separately, in ice phosphate buffer (0.25 M, pH 7.4) in a Potter–Elvehjem type homogenizer. The homogenates were centrifuged at 10,000 Xg for 20 min at 4 °C to pellet the cell debris, and the supernatant was collected and stored at − 80 °C for the determination of NF-κB, caspase-3, TBARS, GSH, GR, GPx, GST, CAT, SOD, and TAC.

### Body and reproductive organs weights

The body weights of rats were recorded. Body weight gain was calculated. Testes, prostates, and epididymis weights were recorded, and % relative testes, prostates, and epididymis weights were calculated as follows: relative testes, prostates, and epididymis weights = (testes, prostates, and epididymis weights/ Final body weight) *100.

### Semen characteristics

The epididymis was prepared for fertility evaluation to evaluate sperm counting, spermatozoa motility parameters, and sperm morphology. Computer-Assisted Semen Analysis (CASA) system (SpermoLyzer^®^, Miralabs) in accordance with the World Health Organization (WHO) 2021 guidelines was used for semen examination with Olympus microscope (Olympus, Tokyo, Japan) according to the method of Adamkovicova et al.^30^. A total of 200 spermatozoa from each rat were examined and individually scored normal or abnormal, according to the strict sperm morphology criteria of Wang et al.^31^.

### Reproductive hormones analysis

Testosterone, Follicle-stimulating hormone (FSH), and Luteinizing hormone (LH) were determined using rat-specific ELISA kits (Cusabio, USA, with Catalog No. CSB-E05100r, CSB-E06869r, and CSB-E12654r, respectively). The assays were performed strictly according to the procedure given along with the kits.

### Apoptotic marker and inflammatory marker in testicular tissues

Caspase-3 has a specificity for cleavage at the terminal side of the aspartate residue of the amino acid sequence DEVD ( Asp-Glu-Val-Asp). The caspase-3 enzymatic activity was assayed using Caspase-3 Assay Kit (Elabscainces, USA, Catalog No: E-CK-A383). Rat Commercial ELISA kits (Chongqing Biospes, China, Catalog No.: BYEK1184) were used for the determination of nuclear factor kappa B (NF-κB) in the testes tissue supernatants according to the manufacturer’s instructions.

### Quantitative analysis of testicular expression of genes controlling mitochondrial biogenesis using real-time PCR

Quantitative analysis of testicular gene expression of PGC-1α, TFAM, and UCP1 was performed using quantitative real-time reverse transcriptase-polymerase chain reaction (qRT-PCR). First, total RNA was isolated from testes using the RNeasy Mini Kit (Qiagen^®^, Germany, Catalog No: 74104) according to the manufacturer’s instructions, and the concentration and integrity of extracted RNA were checked using a nanodrop. Reverse transcription was conducted using the miScript II RT Kit according to the manufacturer’s instructions. The tissue expression of PGC-1α, TFAM, and UCP-1 was quantified in the cDNA using the QuantiTect SYBR Green PCR Kit (Qiagen^®^, USA, Catalog No. 204141). Quantitative PCR amplification conditions were adjusted as an initial denaturation at 95 °C for 10 min and then 45 cycles of PCR for amplification as follows: denaturation at 95 °C for 20 s, annealing at 55 °C for 20 s, and extension at 70 °C for 15 s. The housekeeping gene (18s rRNA) was used as a reference gene for normalization. The primers used for the determination of rat genes are presented in Table 2 (Primers were designed using NCBI Primer-BLAST online tool, http://www.ncbi.nlm.nih.gov/tools/primer-blast). The relative change in mRNA expression in samples was estimated using the 2^−ΔΔCt^ method^32^.

**Table 2:** Primer sets of peroxisome proliferator activator receptor gamma-coactivator 1α, mitochondrial transcription factor A, uncoupling protein 1, and (Reference gene).

Gene	Accession no.	Primer sequence (5’ − 3’)	
18 S rRNA	NR_046237.2	F	GTAACCCGTTGAACCCCATT
		R	CAAGCTTATGACCCGCACTT
PGC-1α	NM_031347.1	F	GTGCAGCCAAGACTCTGTATGG
		R	GTCCAGGTCATTCACATCAAGTTC
TFAM	NM_031326.2	F	CCCTGGAAGCTTTCAGATACG
		R	AATTGCAGCCATGTGGAGG
UCP1	NM_012682.2	F	CAAACAGTTCTACACCAA
		R	CGAAGGCAGAAGTGAAGTTGG

### Testicular oxidative markers and enzymatic and non-enzymatic antioxidants

Thiobarbituric acid reactive substances (TBARS) were measured in testicular homogenates as described by Tappel and Zalkin^33^. Glutathione levels (GSH) were determined by kits obtained from Bio Diagnostic, Egypt, according to the manufacturer’s instructions. Glutathione reductase (GR) (GR; E.C. 1.6.4.2) was determined by the kits obtained from Bio Diagnostic, Egypt, according to the manufacturer’s instructions. Glutathione Peroxidase (GPx) (GPx; EC 1.1.1.9) was determined by the kits obtained from Bio Diagnostic, Egypt, according to the manufacturer’s instructions. Superoxide dismutase (SOD, EC 1.15.1.1) was assayed in plasma according to the method described by Nishikimi et al.^34^. Catalase (CAT; EC 1.11.1.6) was determined according to the method described by Sinha^35^ with some modifications. Total antioxidant capacity (TAC) was assayed in plasma according to the manual instructions of the Bio Diagnostic Kit, Egypt (Catalog No. TA2513).

### Histopathological examination

Immediately after decapitation, animals were dissected, testes from different groups were quickly removed, washed in 0.9 saline solutions, and fixed in 10% neutral buffered formalin. After fixation, specimens were dehydrated in an ascending series of alcohol, cleared in two changes of xylene, and embedded in molten paraffin (mp. 50–58 °C). Sections of 7 microns thickness were cut using a rotary microtome and mounted on clean slides. Sections were stained with Ehrlich’s hematoxylin and counterstained with eosin after Tousson (2016)^36^. The seminiferous tubules were evaluated using the Johnsen score for rats, in which all tubules in one section were examined and given a score from one to 10 according to the degree of germ cell maturity. The mean score was then calculated by dividing the sum of scores by the number of tubules examined^36^.

### Statistical analysis

Data were analyzed using Prism software package version 5 (GraphPad Prism 5.0). The data were expressed as mean ± SD. The Kolmogorov-Smirnov test was used to study the normal distribution of the studied parameters. The analysis of variance (ANOVA) was made and followed by a post hoc (Bonferroni test) to compare the mean values between and within treated groups compared to untreated and control groups. Differences were considered statistically significant at *p*-value < 0.05.

## Results

### Characterization of polystyrene microplastics

**Fig. 1:**
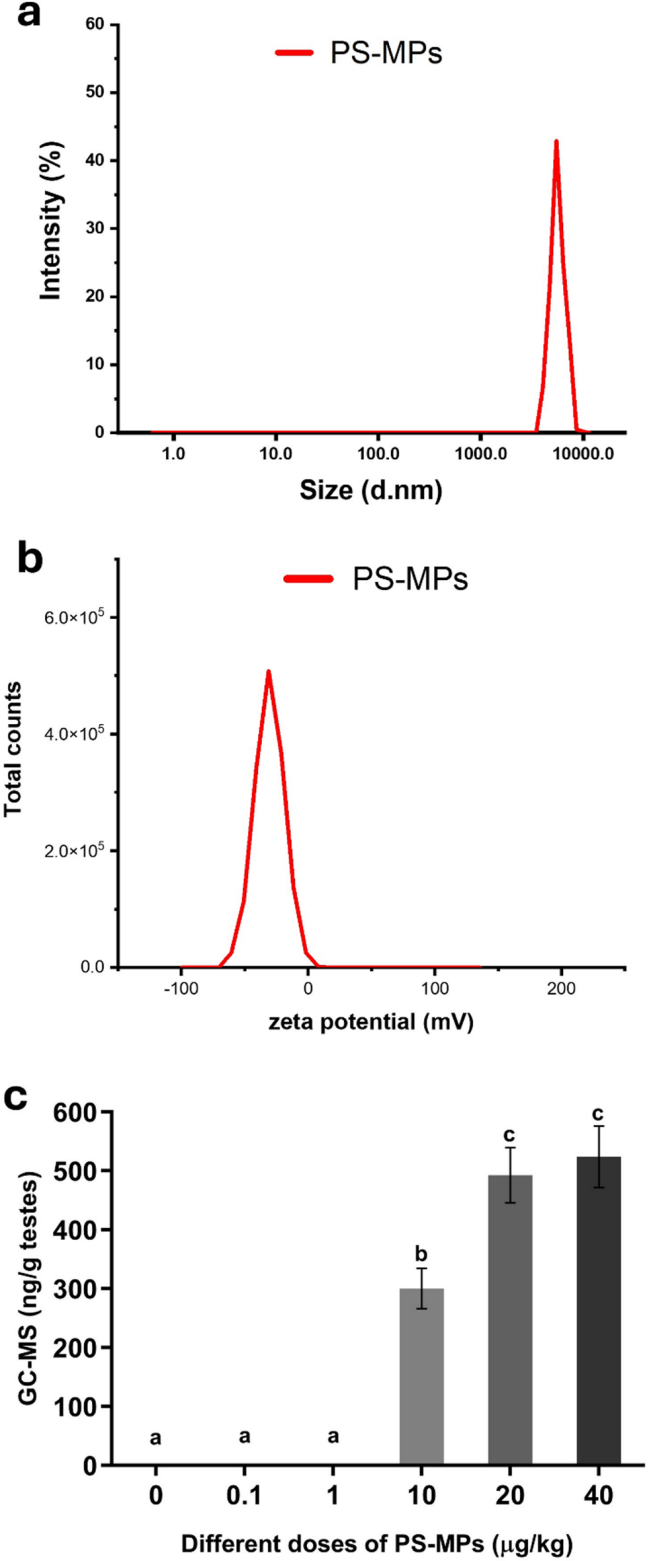
Characterization of PS-MPs by DLS and the detection of PS-MPs in testes by py GC-MS. (**a**) Particle size, (**b**) Zeta potential, (**c**) GC-MS result. Values are expressed as pooled samples of testes of 5 rats in each group. PS-MPs = Polystyrene microplastics, GC-MS = Gas chromatography-mass spectrometry; Value expressed as means ± S.D., *n* = 5. Means with different letters are significantly different, while means with the same letters are not significantly different by ANOVA, followed by Bonferroni post hoc test (*p* < 0.05).

The hydrodynamic diameter and zeta potential of the used PS-MPs are summarized in Fig. 1. The microplastic exhibited particle sizes of about 5200 nm (Fig. 1a) and a zeta potential of about − 31 mV (Fig. 1b).

### The detection of polystyrene microplastics in testes by pyrolysis-gas chromatography-mass spectrometry

The presented data in Fig. 1 showed the changes in testes detection of PS-MPs in adult male rats administered orally with different doses (0.1, 1, 10, 20, and 40 µg/kg BW) of PS-MPs daily for 45 days. The results revealed that PS-MPs were not detected in groups of control, 0.1, and 1 µg/kg BW, while PS-MPs were detected in groups of 10, 20, and 40 µg/kg BW at concentrations of 300.0 ± 34.26, 492.8 ± 46.94, and 524.0 ± 52.48 ng/g tissue, respectively (Fig. 1c).

### Changes in body weight gain and relative sex organ weights of male rats administered with polystyrene microplastics

**Fig. 2:**
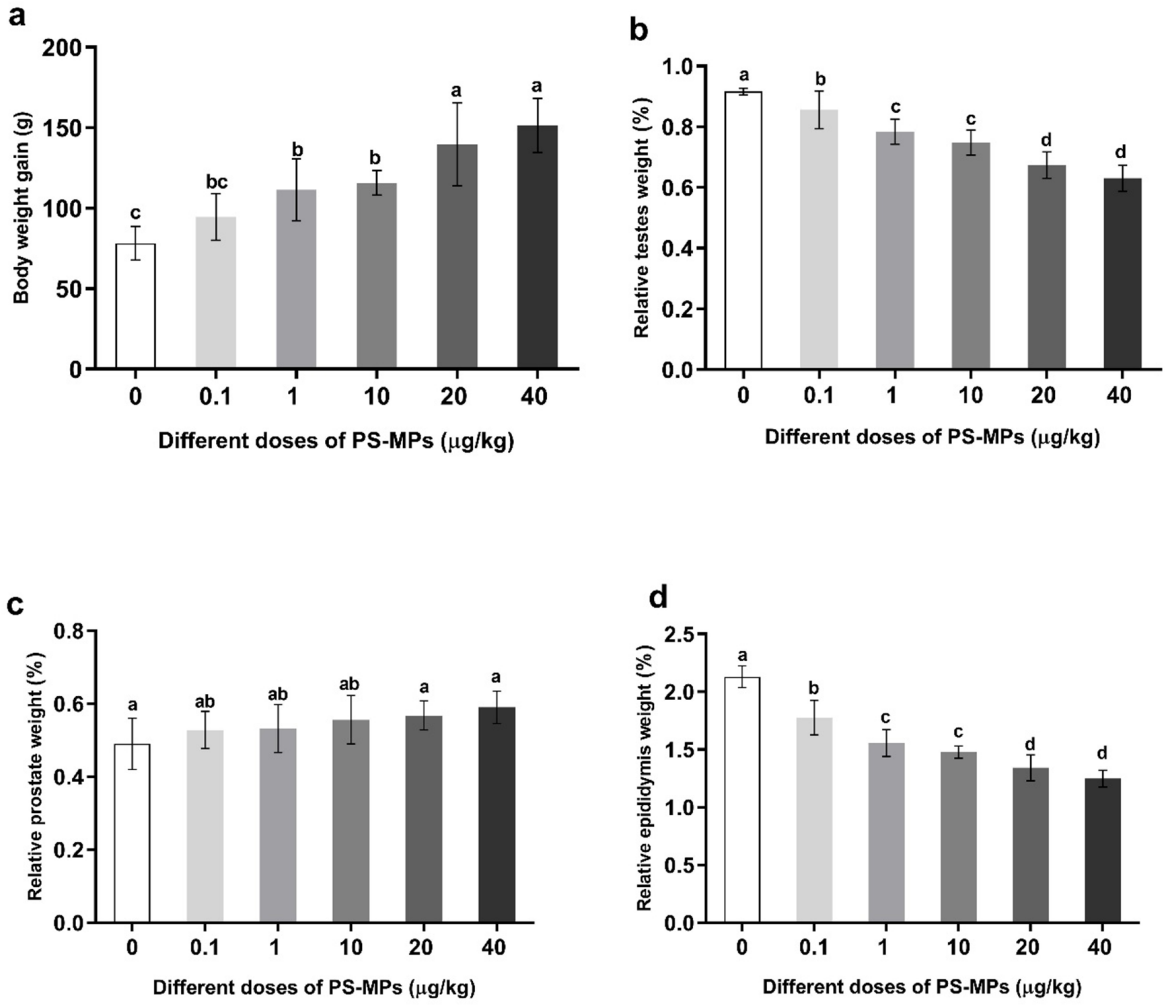
Changes in adult male rats’ body and organ relative weights. (**a**) body weight gain, (**b**) Relative testes weight, (**c**) Relative prostate weight, (**d**) Relative epididymis weight. Value expressed as means ± S.D., *n* = 5. Means with different letters are significantly different, while means with the same letters are not significantly different by ANOVA, followed by Bonferroni post hoc test (*p* < 0.05). R.T.W: Relative testes weight, R.P.W: Relative prostate weight, R.E.W: Relative epididymis weight.

The results showed that body weight gain significantly increased in the groups administered high doses of PS-MPs (10, 20, and 40 µg/kg BW) compared to the control group (Fig. 2a). Relative testes weight and relative epididymis weight significantly decreased in all PS-MPs-administered groups compared to the control group (Fig. 2b and d, respectively). Conversely, relative prostate weight increased significantly in all PS-MPs-administered groups relative to the control group (Fig. 2c).

### Changes in sex hormones and semen characteristics of male rats administered with polystyrene microplastics

The current study investigated the effects of orally administered PS-MPs on the reproductive parameters of adult male rats. Specifically, it assessed alterations in sex hormones (testosterone, follicle-stimulating hormone (FSH), and luteinizing hormone (LH)), and semen characteristics (sperm count, motility, and morphology). Rats were administered PS-MPs at varying doses (0.1, 1, 10, 20, and 40 µg/kg BW).

**Fig. 3:**
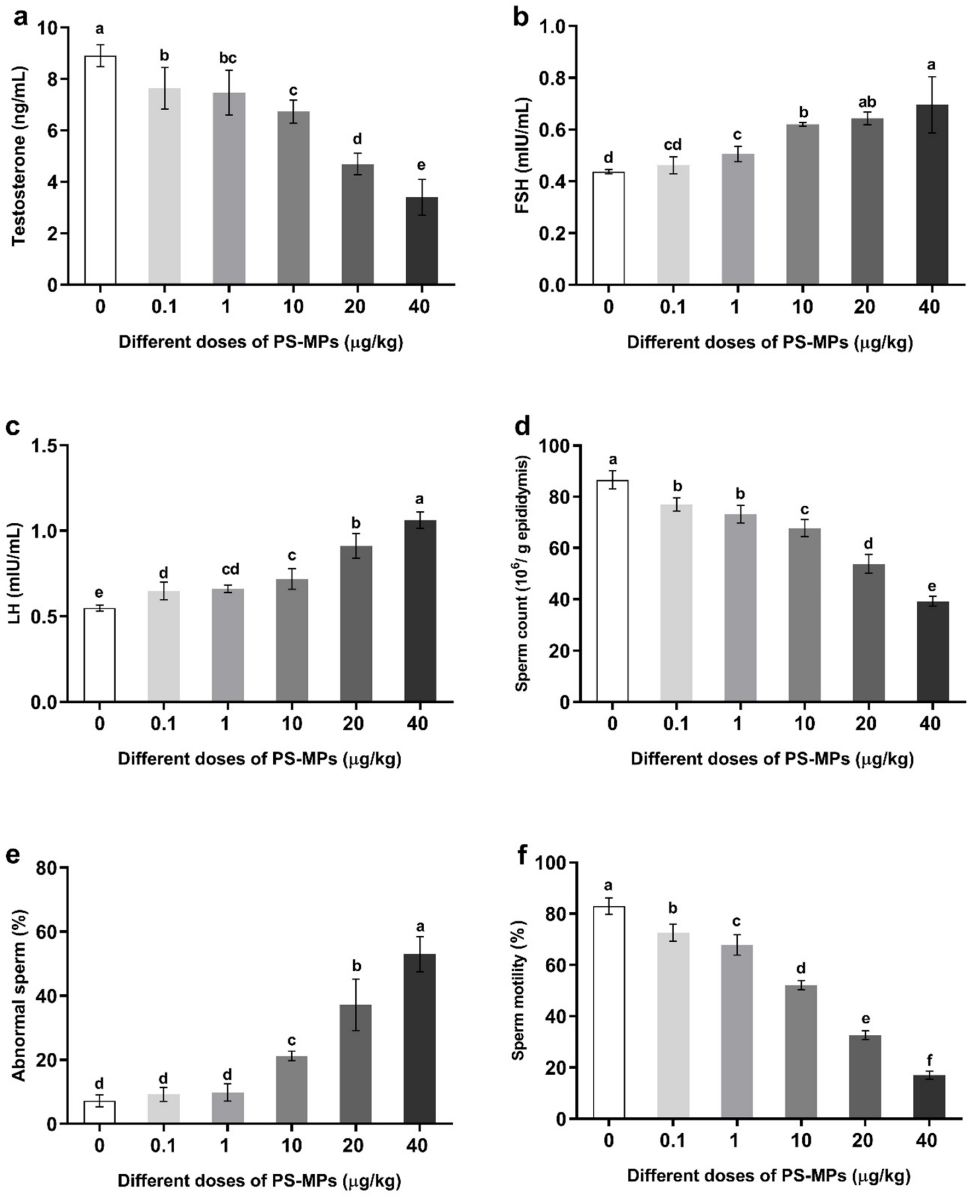
Changes in reproductive hormones in plasma (**a**) Testosterone, (**b**) FSH, (**c**) LH, and semen characteristics; (**d**) Sperm count, (**e**) Abnormal sperm, (**f**) Sperm motility. Value expressed as means ± S.D., *n* = 5. Means with different letters are significantly different, while means with the same letters are not significantly different by ANOVA, followed by Bonferroni post hoc test (*p* < 0.05). FSH: Follicle-stimulating hormone, LH: Luteinizing hormone.

The data demonstrated a significant dose-dependent decline in Testosterone levels across all treatment groups compared to the control group. Conversely, FSH and LH levels exhibited a significant dose-dependent increase in all PS-MP-administered groups (Fig. 3a and b, and 3c).

Furthermore, the results indicated that both sperm count and sperm motility significantly decreased in a dose-dependent manner (Fig. 3d and f). In contrast, the percentage of abnormal sperm showed a significant dose-dependent increase in all treated groups relative to the control group (Fig. 3e).

### Changes in oxidative stress of male rats administered with polystyrene microplastics

**Fig. 4:**
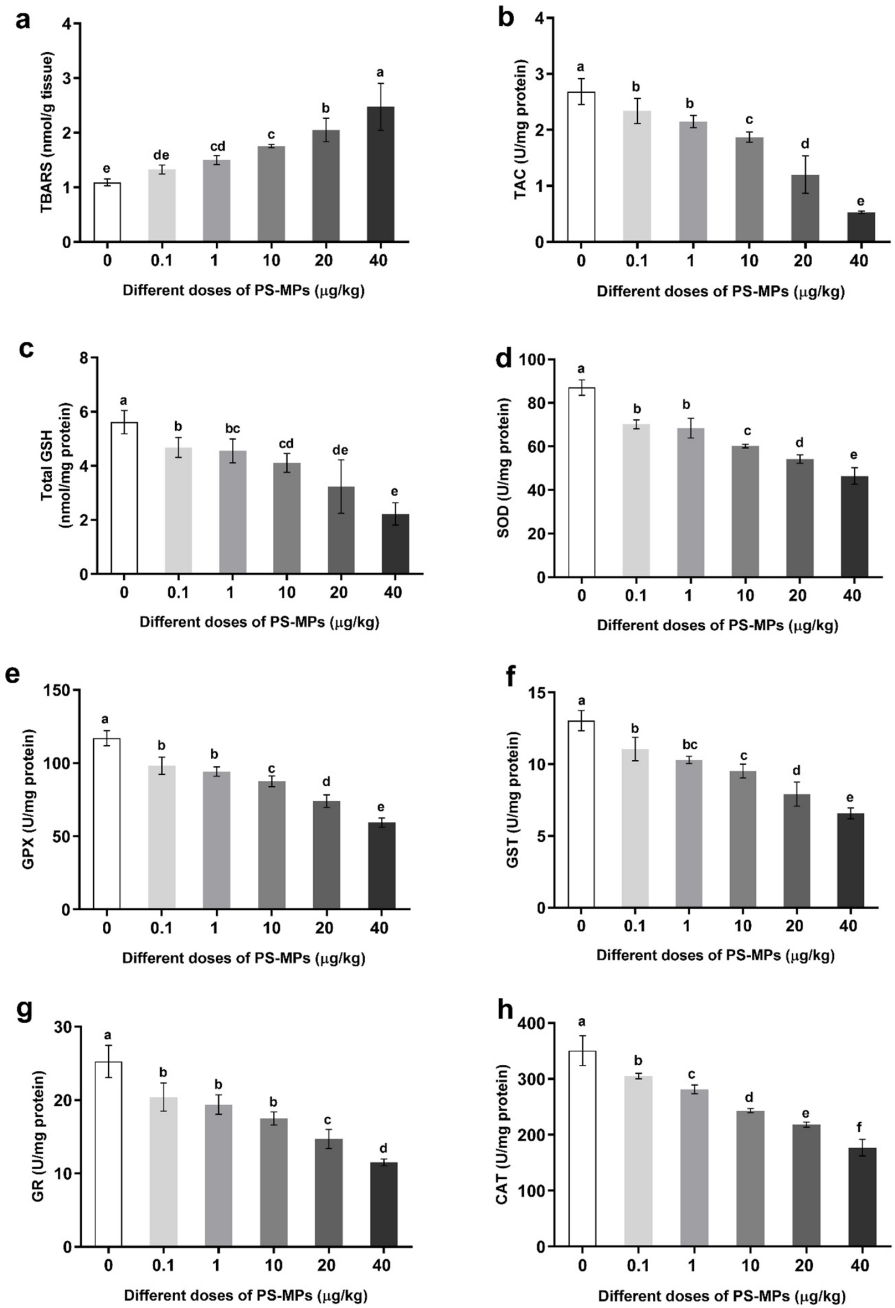
Changes in plasma oxidative stress, enzymatic and non-enzymatic antioxidant parameters; (**a**) TBARS, (**b**) TAC, (**c**) GSH, and enzymatic and non-enzymatic antioxidants; (**d**) SOD, (**e**) GPX, (**f**) GST, (**g**) GR, and (**h**) CAT. Value expressed as means ± S.D., *n* = 5. Means with different letters are significantly different, while means with the same letters are not significantly different by ANOVA, followed by Bonferroni post hoc test (*p* < 0.05). TBARS = Thiobarbituric acid-reactive substances, TAC = Total antioxidant capacity, and GSH = Glutathione levels, SOD: Superoxide dismutase, GPx: Glutathione peroxidase, GST: Glutathione S-transferase, GR: Glutathione reductase, and CAT: Catalase.

The results revealed increased oxidative stress levels in adult male rats following oral administration of varying doses of polystyrene microplastics (PS-MPs) (0.1, 1, 10, 20, and 40 µg/kg BW). Specifically, the thiobarbituric acid-reactive substances (TBARS) level showed a significant dose-dependent increase in all PS-MP-treated groups compared with the control group (Fig. 4a).

### Changes in the enzymatic antioxidants of male rats administered with polystyrene microplastics

The results revealed a significant, dose-dependent decrease in the activities of the antioxidant enzymes superoxide dismutase (SOD), glutathione peroxidase (GPX), glutathione S-transferase (GST), glutathione reductase (GR), and catalase in all groups administered with PS-MPs when compared to the control group (Figs. 4d–h).

### Changes in the non-enzymatic antioxidants of male rats administered with polystyrene microplastics

The results indicated that glutathione levels (GSH) and total antioxidant capacity (TAC) showed a significant dose-dependent decrease in all groups administered with PS-MPs compared with the control group, as shown in (Fig. 4b, c).

### Changes in testes peroxisome proliferator activator receptor gamma-coactivator 1α expression, mitochondrial transcription factor A expression, and uncoupling protein 1 expression of male rats administered with polystyrene microplastics

**Fig. 5:**
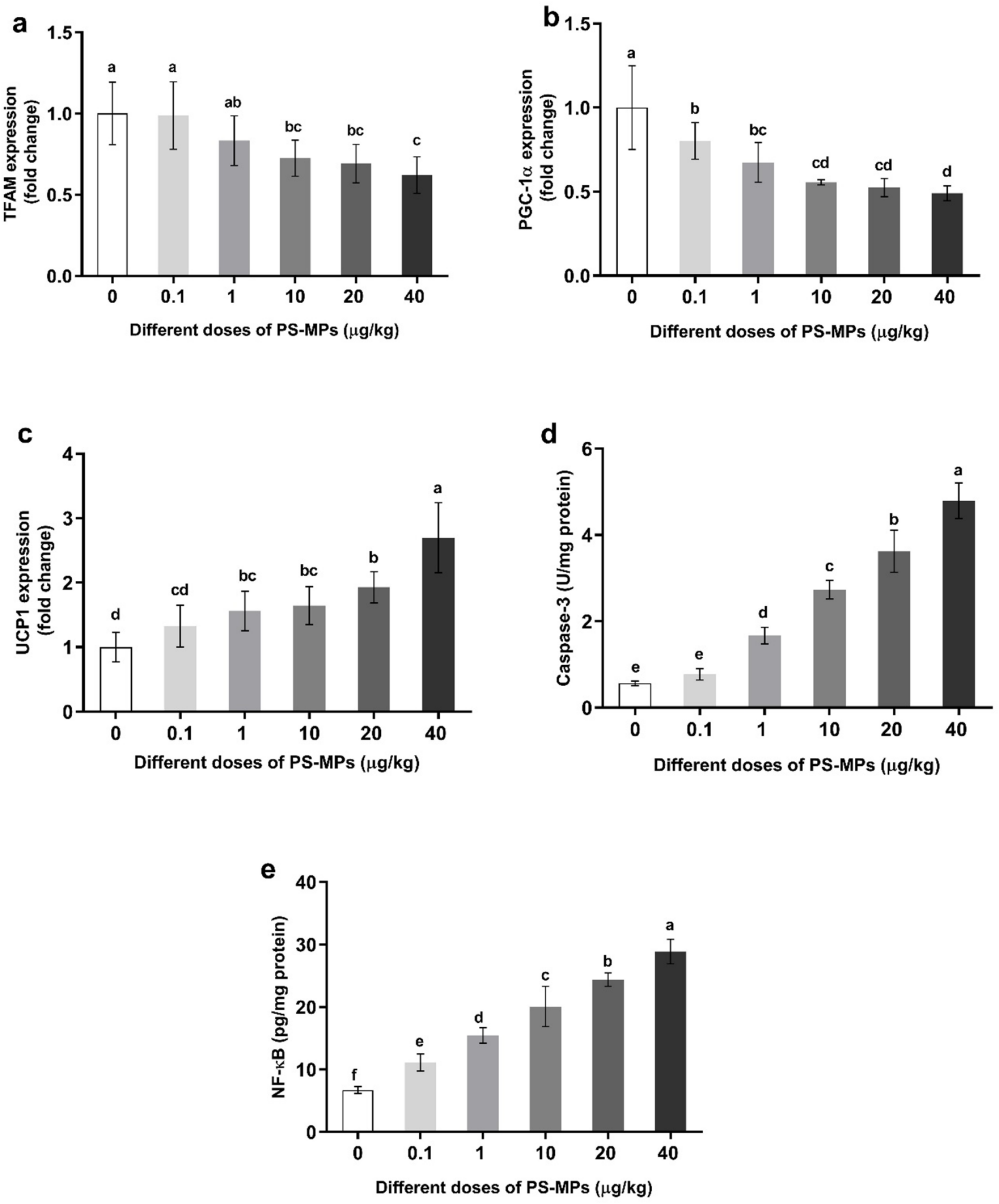
Changes in testes mitochondrial function, apoptotic, and inflammatory markers; (**a**) TFAM, (**b**) PGC-1α, (**c**) UCP1, (**d**) caspase-3 activity, (**e**) NF-κB. Value expressed as means ± S.D., *n* = 5. Means with different letters are significantly different, while means with the same letters are not significantly different by ANOVA, followed by Bonferroni post hoc test (*p* < 0.05). TFAM: Mitochondrial transcription factor A, PGC-1α: Peroxisome proliferator-activated receptor gamma-coactivator 1α, UCP1: Uncoupling protein 1, NF-κB = Nuclear factor kappa B.

The data from the present study (Fig. 5a-c) illustrate the dose-dependent changes in the expression of TFAM, PGC-1α in the testes, and UCP1 in adult male rats. These rats were orally administered polystyrene microplastics (PS-MPs) at various doses (0.1, 1, 10, 20, and 40 µg/kg BW).

The results obtained reveal a consistent pattern across all PS-MP-administered groups compared to the control: PGC-1α and TFAM expression significantly decreased, whereas UCP1 expression significantly increased. These alterations were observed to follow a clear dose-dependent manner.

### Changes in testis apoptotic marker, Caspase-3 activity of male rats administered with polystyrene microplastics

Figure 5d presents the changes in testicular caspase-3 activity, an established apoptotic marker, in adult male rats following oral administration of various doses of PS-MPs(0.1, 1, 10, 20, and 40 µg/kg BW). The data indicate a significant and dose-dependent increase in caspase-3 activity across all PS-MP treatment groups compared to the control group.

### Changes in testis inflammatory marker, nuclear factor kappa B, of male rats administered with polystyrene microplastics

Figure 5e presents the changes in the testicular concentration of NFκB, an inflammatory marker, in adult male rats following oral administration of various doses (0.1, 1, 10, 20, and 40 µg/kg BW) of PS-MPs. The results indicated a significant, dose-dependent increase in NFκB content across all PS-MP-treated groups compared to the control group.

### Testis histopathology

**Fig. 6:**
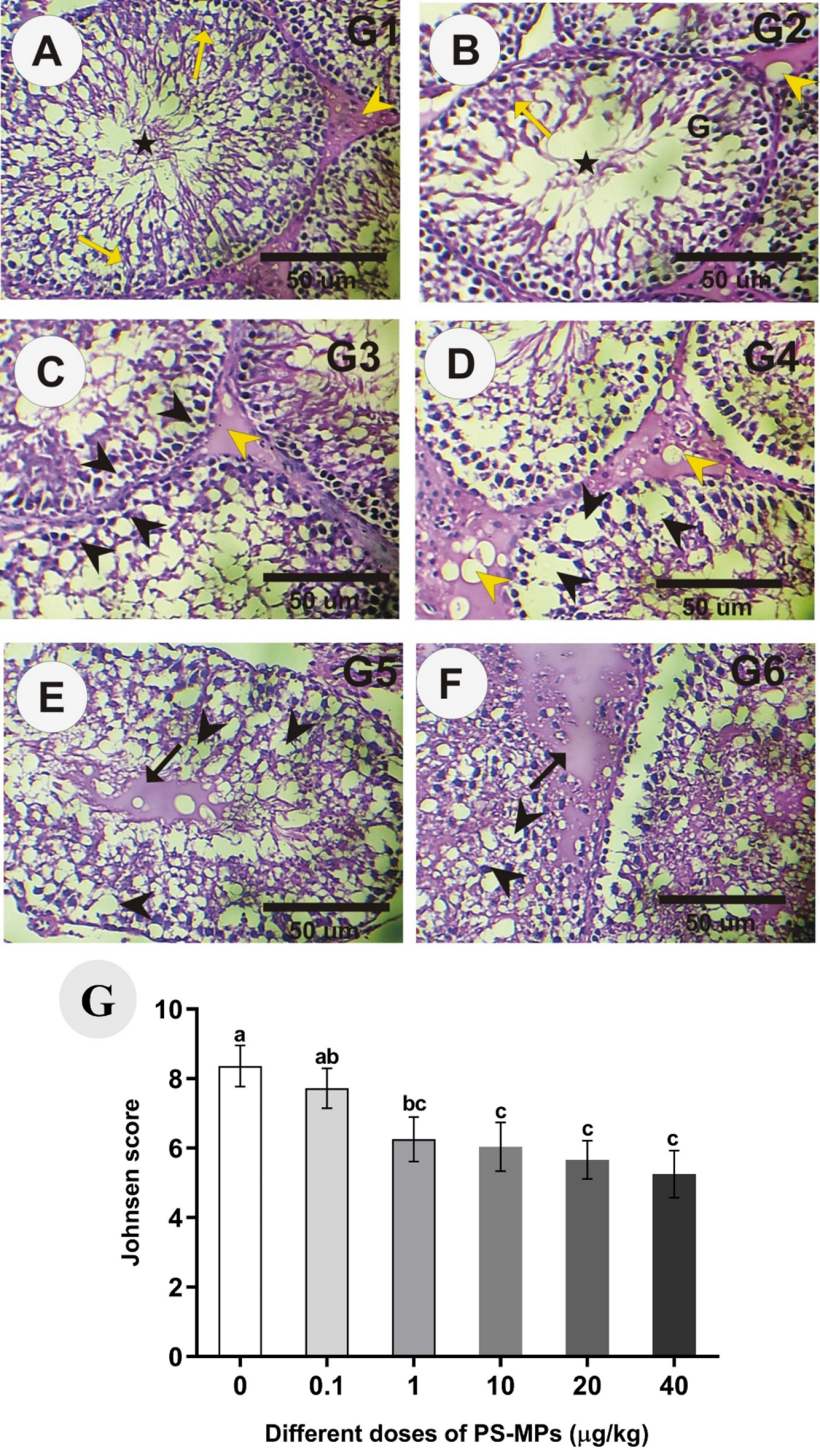
Photomicrographs of rat testes sections stained by HE. A&B: Rat testes in the control **(A)** group showed a normal structure of seminiferous tubules that were fully packed with sperm (stars). (**B**,**C**): Rat testes in 0.1 and 1.0 µg/kg treated groups revealed mild to moderate morphological changes, such as moderate vacuolar degenerative changes in the cytoplasm of the spermatogenic epithelium, although the seminiferous tubules (Black arrow heads) were semi-full packed with sperms, and a mild decrease in Leydig cells (Yellow arrow heads). **(D)**: Testes sections in the 10 µg/kg treated group revealed moderate irregularity and disturbance in spermatogenesis cycles, moderate vaculation (Black arrow heads), and a significant increase in Leydig cells (Yellow arrow heads). **(E)**: Testes sections in the 20 µg/kg treated group revealed marked irregularity and disturbance in spermatogenesis cycles with marked morphological changes, such as marked vacuolar degeneration of germinal epithelium (Black arrow heads) and a significant marked decrease in the number of sperm cells (Black arrows). (**F**) Testes sections in the 40 µg/kg treated group revealed severe vacuolar degeneration of germinal epithelium (Black arrow heads), marked decrease in the number of spermatogenic cells in the seminiferous tubules, and absence of sperms (Black arrows). (**G**) Comparison between the different groups regarding the Johnsen score; Value expressed as means ± S.D., *n* = 5. Means with different letters are significantly different, while means with the same letters are not significantly different by ANOVA, followed by Bonferroni post hoc test (*p* < 0.05).

Figure 6 revealed the histopathological changes and the Johnsen score in the testes in different groups. Histopathological study showed that the cycle of spermatogenesis was regular in all male rats in control (Fig. 1A). The structural components of the testis were the seminiferous tubules and interstitial cells known as Leydig cells. Two types of cells were identified in rat seminiferous tubules: the Sertoli cells and the spermatogenic cells. Sertoli cells were found resting on the thin basal lamina (basement membrane) while the spermatogenic cells were arranged in many layers (spermatogonia, primary spermatocytes, secondary spermatocytes, spermatids, and sperms). The lumen in the seminiferous tubules in control rats revealed full packed with sperms (Fig. 1A). However, the light microscopy examination of the testes of the 0.1 and 1.0 µg/kg treated groups revealed abnormal disturbance in spermatogenesis cycles with mild to moderate morphological changes such as moderate vacuolar degenerative changes in the cytoplasm of the spermatogenic epithelium although the seminiferous tubules were semi full packed with sperms, and mild decrease in Leydig cells (Fig. 1B and C). Testes sections in the 10 µg/kg treated group revealed moderate irregularity and disturbance in spermatogenesis cycles, moderate vaculation, moderate a significant marked decrease in the number of spermatogenic cells, mild degenerative changes were also appeared in the cytoplasm of the spermatogenic epithelium, mild hemorrhage, and a moderate significant decrease in Leydig cells as compared with 0.1 and 1.0 µg/kg treated groups (Fig. 1D).

Testes sections in the 20 µg/kg treated group revealed marked irregular and disturbance in spermatogenesis cycles with marked morphological changes such as marked vacuolar degeneration of germinal epithelium and marked a significant marked decrease in the number of spermatogenic cells while testes sections in the 40 µg/kg treated group revealed the presence of many of a syncytial cells, marked hemorrhage, sloughing of germ cells into the tubular lumen, a significant marked decrease in the number of spermatogenic cells in the seminiferous tubules and deficit and very little Leydig cell numbers (Fig. 1E and F respectivelly).

## Discussion

The study provides solid evidence of the dose-dependent reproductive toxicity of oral exposure to polystyrene microplastics (PS-MPs) at doses ranging from 0.1 to 40 µg/kg body weight, in male rats. Our findings demonstrate that PS-MPs cause severe adverse effects on testicular architecture and fertility, characterized by a decline in semen quality and a significant disruption of sex hormone balance. PS-MPs trigger inflammation and apoptosis, evidenced by increased NF-κB and caspase-3 expression, and impair mitochondrial function by altering the expression of PGC-1α, TFAM, and UCP1. This disruption of cellular processes leads to significant oxidative stress, as shown by elevated TBARS and depleted antioxidant reserves (GSH, GR, GPx, SOD, CAT, and TAC). The direct presence of PS-MPs in the testes was confirmed at higher doses (10, 20, and 40 µg/kg), solidifying the link between exposure and the observed testicular damage.

Human exposure models suggest that typical adult intake of microplastics is on the order of micrograms to low milligrams per day, corresponding roughly to about 0.01–0.7 mg/kg/day depending on scenario and plastic type^37^. while experimental environmentally relevant PS‑MP doses used in rodents are often in the range around 2.7–8.1 mg/kg/day derived by scaling from estimated human intake, or single test doses of 8 mg/kg/day, whereas other toxicology studies have used higher doses such as 25–30 mg/kg/day or more to probe hazard under worst‑case exposure^38–40^. In the present study, rats received 0.1–40 µg/kg/day PS‑MPs by oral gavage, i.e., doses that are 10–1000‑fold lower than the mg/Kg ranges frequently employed in rodent microplastic toxicology studies.​​These µg/kg doses fall within or below upper‑bound estimates of human and wildlife microplastic exposure. The current investigation clarified the bioaccumulation and toxicity of these low doses of PS-MPs in the testicular tissues of male rats. We used pyrolysis gas chromatography-mass spectrometry (Py-GC-MS) for PS-MPs detection in testicular tissue due to their ability to detect MPs of any size without prior separation^41^. This method has been effective in studying PS-MPs ingested by male rats, especially in the presence of an organic alkali^42^. Inconsistent with the previous research, Py-GC-MS results revealed that PS-MPs were detected at doses of 10 µg/kg and higher. Py-GC-MS analysis revealed styrene monomer and trimer peaks from polystyrene, with the amount of PS linearly correlating to peak area.

Many animal studies have investigated the association between MPs and reproductive dysfunction, particularly in males; however, the molecular mechanisms have yet to be determined. At histological level, the testes of rats treated with increasing low-does of PS-MPs (0.1–40 µg/kg) showed dose-dependent architectural changes as irregular and disturbed spermatogenesis cycles, vacuolar degeneration, degenerative alterations in the spermatogenic epithelium’s cytoplasm, hemorrhage, reduction in the number of spermatogenic cells in the seminiferous tubules, a decline in Leydig cell numbers, an extensive amount of syncytial cells. Similarly, previous studies showed that PS-MPs treatment using higher doses disrupts spermatogenesis, causing irregular arrangement of spermatogenic stages, reduces sperm motility, viability, and normal morphology, while increasing sperm malformations and dead cells^43–45^. Furthermore, the current study and earlier studies found that exposure to PS-MP raises body weight and reduces the relative testicular and epididymal weights in a dose-dependent manner, indicating poor reproductive organ development^27,43^. Testicular weight loss is linked to suppressed spermatogenesis, testicular hypocellularity, and seminiferous tubule atrophy, consistent with observed changes in testis histology, semen quality, and reproductive hormone levels^46^.

In line with previous studies, the oral exposure to PS-MPs dose-dependently reduces sperm motility and count but increases sperm deformities in male rats^47^. These morphological and quantitative seminal abnormalities are directly linked to the testicular pathology: PS-MPs cause rupture, nuclear degeneration, and shedding of spermatogonia in the seminiferous tubules, ultimately harming sperm quantity and quality^48,49^. These profound abnormalities were associated with perturbations in sex hormone levels, suggesting PS-MPs function as significant endocrine disruptors through their interference with the hypothalamic-pituitary-gonadal (HPG) axis.

Normally, gonadotropin-releasing hormone (GnRH) from the hypothalamus stimulates pituitary secretion of LH and FSH, which act on Leydig and Sertoli cells, respectively, to support testosterone production and spermatogenesis^50^. Testosterone (from Leydig cells) and inhibin (from Sertoli cells) then exert negative feedback on the hypothalamus and pituitary, suppressing GnRH, LH, and FSH secretion and keeping the axis in balance^51^. In the context of PS-MPs, this feedback loop is disrupted: the data show reduced testosterone together with histological Leydig cell loss and impaired spermatogenesis, indicating primary testicular failure rather than central (hypothalamic–pituitary) hypogonadism.​​ As testosterone levels fall, negative feedback on the hypothalamus and pituitary weakens; in response, GnRH secretion and pituitary output of LH and FSH increase in a compensatory attempt to stimulate the failing testes, leading to the observed hypergonadotropic hypogonadism pattern (high LH/FSH with low testosterone). Similar endocrine profiles have been described after PS‑MP exposure in rodent models, where MPs impair Leydig cell steroidogenesis and downregulate LH‑mediated signaling pathways^22,27,43,52–56^. Thus, the concurrent decrease in testosterone, dose‑dependent elevation of LH and FSH, and structural Leydig/Sertoli cell damage in the present study support the interpretation that PS‑MPs primarily injure the testes, and that the HPG axis responds with compensatory gonadotropin upregulation that is insufficient to restore normal androgen production.

The exposure to PS-MPs has been shown to induce oxidative stress in murine testes, leading to reduced sperm count and activation of the p38 MAPK signaling pathway, both of which negatively impact fertility^9,22^. These are in agreement with the current study, which observed a significant dose-dependent elevation of testicular TBARS (lipid peroxidation end products), associated with a significant decline in antioxidant systems, including total antioxidant capacity, GSH content, and the activities of CAT, GPX, GR, GST, and SOD. These abnormalities are significant at very low doses (about 1 µg/kg) for most parameters. The MPs-induced oxidative stress and toxicity are dependent on the exposure times and MPs; the longer time and smaller size (bigger surface area), the more exaggerated the oxidative stress^57–59^. In agreement with our research, mice exposed to MPs showed decreased CAT activity^60^. According to Wei et al., ROS and MDA levels in the testes were significantly elevated after being exposed to PS-MPs, while dramatically lowering the levels of GSH. Furthermore, PS-MPs decreased the activity of the antioxidant enzymes: peroxidase, SOD, CAT, GSH, and TAC while elevating the level of lipid peroxidation and ROS^39,61^. The resulting imbalance between the body’s oxidation and antioxidant defense mechanisms plays a crucial role in male infertility.

The testicular oxidative stress induced by low doses of PS-MPs was associated with a proinflammatory status, as indicated by a marked dose-dependent elevation of NF-κB, an essential regulator of PS-MPs-induced inflammation in testicular tissue^9^. Oxidative stress and inflammation are linked pathophysiological processes that mutually induce one another in the context of male infertility^9^. Inflammation in the male reproductive tract activates immune cells, as confirmed by histological findings, which in turn exacerbate oxidative stress through the burst of excess ROS generation. ​Conversely, ROS can activate intracellular signaling cascades that promote the activation of proinflammatory genes, which creates a vicious cycle^62^.

In our study, at the mitochondrial level, the oral exposure to low doses of PS-MPs induced a significant downregulation of the expression of, PGC-1α and TFAM, while significantly upregulating the expression of UCP1. Thee results align with previous research, indicating that PS-MPs induced mitochondrial damage and energy metabolism disorders through the downregulation of TFAM^63^. PGC‑1α and TFAM are key regulators of mitochondrial biogenesis; their suppression in testicular tissue suggests reduced mitochondrial mass and impaired maintenance of the mitochondrial network in germ cells^64,65^. During spermatogenesis and in mature spermatozoa, mitochondria localized in the midpiece are the primary source of ATP required for flagellar movement, so disturbances in mitochondrial biogenesis and function are tightly linked to reduced sperm motility and abnormal morphology^66^. The combination of elevated lipid peroxidation (TBARS) and depleted antioxidant systems indicates excessive mitochondrial ROS production, which can damage mitochondrial membranes and mtDNA, further lowering ATP generation and exacerbating sperm functional impairment^67^.

Overexpression of UCP1 may represent an adaptive response to limit ROS by uncoupling oxidative phosphorylation, but this also promotes proton leak and ATP depletion, providing a plausible mechanistic explanation for the observed decline in sperm motility and counts^68,69^. In line with these, PS-MPs can disrupt mitochondrial dynamics and biogenesis in mouse testes, and Sertoli cells which can trigger mitochondria-mediated apoptosis^70^. The induced apoptosis in testicular tissues was detected in the present study as a significant activation of caspase-3 activities (a pivotal effector in the apoptotic pathways) in a dose-dependent manner. The ROS overload disrupts the electron transport chain in mitochondria, leading to ATP depletion and activation of apoptotic pathways in Leydig and Sertoli cells^71–73^. These findings align with Ijaz et al.^39^, who reported a dose-dependent increase in caspase-3 activity in rats exposed to PS-MPs compared to controls, but using higher doses and for a longer period of time (60 days).

Taken together, our findings support a model in which PS‑MPs dose-dependently accumulate in the testis and trigger oxidative stress, leading to mitochondrial biogenesis impairment (PGC‑1α/TFAM downregulation), mitochondrial uncoupling (UCP1 upregulation), and activation of mitochondria‑dependent apoptosis (caspase‑3), ultimately compromising spermatogenesis and Leydig cell steroidogenesis and resulting in reduced sperm quality and testosterone deficiency.

While the present study provides a comprehensive assessment of PS-MPs-induced reproductive toxicity at low doses, certain limitations should be acknowledged. The small group size reduces statistical power to detect subtle effects, especially at low doses, and may increase the likelihood of Type II errors (false negatives), so some smaller true effects might have remained undetected. The mitochondrial membrane potential and ATP content in testicular tissue or spermatozoa were not directly measured; therefore, the proposed link between PGC‑1α/TFAM suppression, mitochondrial uncoupling, and ATP deficiency remains inferential and need future work to directly assess mitochondrial membrane potential, mitochondrial ROS, and ATP levels in sperm and testicular cells to confirm the contribution of mitochondrial bioenergetic failure to PS‑MP‑induced sperm dysfunction. Also, future studies using different types of PS-MPs (size and type) and for a longer period of time are required to provide a more complete risk assessment. Also, investigating the potential for transgenerational effects and the impact of PS-MPs on female reproductive health are critical areas for future studies.

## Conclusion

In conclusion, sub‑chronic oral exposure to low doses (microgram/Kg) of PS-MPs impaired semen quality, disrupted sex hormones, and induced marked testicular histopathological damage in adult male rats. The concurrent oxidative stress, suppression of mitochondrial biogenesis markers (PGC‑1α, TFAM), UCP1 upregulation, and caspase‑3 activation support a unified mechanism whereby PS‑MPs trigger oxidative stress‑driven mitochondrial dysfunction and apoptosis, compromising spermatogenesis and Leydig‑cell steroidogenesis. Given that these doses are within the order of magnitude of environmental exposure estimates, the findings raise concerns about potential impacts on male fertility, although the lack of direct mitochondrial energetics measurements and the sub‑chronic, single‑sex design remain important limitations requiring further investigation.

## Data Availability

All data produced in this study can be made available from the corresponding author upon a reasonable request.
